# 3D CT-Inclusive Deep-Learning Model to Predict Mortality, ICU Admittance, and Intubation in COVID-19 Patients

**DOI:** 10.1007/s10278-022-00734-4

**Published:** 2022-11-30

**Authors:** Alberto Di Napoli, Emanuela Tagliente, Luca Pasquini, Enrica Cipriano, Filomena Pietrantonio, Piermaria Ortis, Simona Curti, Alessandro Boellis, Teseo Stefanini, Antonio Bernardini, Chiara Angeletti, Sofia Chiatamone Ranieri, Paola Franchi, Ioan Paul Voicu, Carlo Capotondi, Antonio Napolitano

**Affiliations:** 1 Radiology Department, Castelli Hospital, 00040 Ariccia, Italy; 2 Medical Physics Department, Bambino Gesù Children’s Hospital, Scientific Institute for Research, Hospitalization and Healthcare (IRCCS), 00165 Rome, Italy; 3 NESMOS Department, Neuroradiology Unit, Sant’Andrea Hospital, Sapienza University, Via Grottarossa 1035, 00189, 00165 Rome, Italy; 4 Radiology Department, Neuroradiology Service, Memorial Sloan Kettering Cancer Center, New York, NY 1275 USA; 5 COVID Medicine Department, Castelli Hospital, 00040 Ariccia, Italy; 6 COVID Intensive Care Unit, Castelli Hospital, 00040 Ariccia, Italy; 7 Emergency Department, Castelli Hospital, 00040 Ariccia, Italy; 8 Radiology Department, Sant’Andrea Civil Hospital, 19121 La Spezia, Italy; 9 Radiology Department, Giuseppe Mazzini Civil Hospital, 64100 Teramo, Italy; 10 Anestesiology, Intensive Care and Pain Medicine, Emergency Department, Giuseppe Mazzini Civil Hospital, 64100 Teramo, Italy; 11 Department of Clinical Pathology, Giuseppe Mazzini Civil Hospital, 64100 Teramo, Italy

**Keywords:** COVID-19, Chest CT, Artificial intelligence, Deep learning, External validation

## Abstract

**Abstract:**

Chest CT is a useful initial exam in patients with coronavirus disease 2019 (COVID-19) for assessing lung damage. AI-powered predictive models could be useful to better allocate resources in the midst of the pandemic. Our aim was to build a deep-learning (DL) model for COVID-19 outcome prediction inclusive of 3D chest CT images acquired at hospital admission. This retrospective multicentric study included 1051 patients (mean age 69, SD = 15) who presented to the emergency department of three different institutions between 20th March 2020 and 20th January 2021 with COVID-19 confirmed by real-time reverse transcriptase polymerase chain reaction (RT-PCR). Chest CT at hospital admission were evaluated by a 3D residual neural network algorithm. Training, internal validation, and external validation groups included 608, 153, and 290 patients, respectively. Images, clinical, and laboratory data were fed into different customizations of a dense neural network to choose the best performing architecture for the prediction of mortality, intubation, and intensive care unit (ICU) admission. The AI model tested on CT and clinical features displayed accuracy, sensitivity, specificity, and ROC-AUC, respectively, of 91.7%, 90.5%, 92.4%, and 95% for the prediction of patient’s mortality; 91.3%, 91.5%, 89.8%, and 95% for intubation; and 89.6%, 90.2%, 86.5%, and 94% for ICU admission (internal validation) in the testing cohort. The performance was lower in the validation cohort for mortality (71.7%, 55.6%, 74.8%, 72%), intubation (72.6%, 74.7%, 45.7%, 64%), and ICU admission (74.7%, 77%, 46%, 70%) prediction. The addition of the available laboratory data led to an increase in sensitivity for patient’s mortality (66%) and specificity for intubation and ICU admission (50%, 52%, respectively), while the other metrics maintained similar performance results. We present a deep-learning model to predict mortality, ICU admittance, and intubation in COVID-19 patients.

**Key Points:**

• 3D CT-based deep learning model predicted the internal validation set with high accuracy, sensibility and specificity (> 90%) mortality, ICU admittance, and intubation in COVID-19 patients.

• The model slightly increased prediction results when laboratory data were added to the analysis, despite data imbalance. However, the model accuracy dropped when CT images were not considered in the analysis, implying an important role of CT in predicting outcomes.

**Supplementary Information:**

The online version contains supplementary material available at 10.1007/s10278-022-00734-4.

## Introduction

The use of artificial intelligence (AI) techniques in the medical field has increased exponentially in recent years [1], with promising results in terms of diagnostic accuracy of predictive models based on machine learning and deep-learning (DL) algorithms [2–4]. Especially in the field of radiology, biomedical images proved to be an optimal input for AI-powered models thanks to the large amount of data available on picture archiving and communication systems (PACS) which can provide new information to help prognostication, given enough computational power [1].

In 2020/2021, the coronavirus disease 2019 (COVID-19) pandemic, caused by severe acute respiratory syndrome coronavirus 2 (SARS-CoV-2), affected the lives of people all over the world with more than 450 million infections and more than 6 million deaths in over 2 years [1]. One of the major problems related to COVID-19 was the difficulty of estimating patients’ prognosis in a context of sanitary resources shortage. The need of correctly allocating the available resources to guarantee the best treatment possible, with the possibility of rationing care delivery, carries important logistical and ethical implications, which have been pointed out as a crucial limitation of healthcare systems dealing with widespread emergencies [5]. The availability of an accurate AI-powered predictive model of patient outcome may help achieving fast diagnosis, optimizing resources, and tailor treatment in these scenarios. AI models were recently tested to predict COVID-19 outcome using clinical and laboratory data, with good accuracy [6]. Chest CT is considered a useful tool in COVID-19 assessment, for its high sensitivity in detecting typical signs of “ground-glass” opacities, crazy paving pattern, and lung consolidations even in subjects with false negative RT-PCR tests (considered the gold standard) [7, 8]. Other CT findings, such as pleural and pericardial effusion, and pulmonary embolism in enhanced chest CT, have been also described [9, 10]. Disease severity on chest CT correlated with the clinical status of COVID-19 patients and was successfully used to predict short-term progression in recent studies [11, 12]. Chest CT-based DL models demonstrated high accuracy in differentiating COVID-19 from community-acquired pneumonia and non-COVID-19-related ground-glass opacities [13, 14].

With the above-written premises, this multicentric study aims to obtain an accurate predictive 3D CT-inclusive model for the outcome of patients affected by COVID-19 by employing artificial neural networks on chest CT images obtained at the first access to the emergency department. We aimed at predicting COVID-19 outcomes from a heterogeneous population including patients from different regions of Italy and images obtained with different scanners. Our hypothesis was that the integration of CT images, clinical, and lab data would achieve high accuracy in COVID-19 outcome prediction in our model.

## Methods

### Patients

We retrospectively evaluated patients who accessed the emergency department at three Italian hospitals from March 2020 to February 2021. The study was approved by the local ethical committee and patients received written informed consent. In case of patients’ inability, informed consent was received from the relatives. Data collection and usage were compliant with General Data Protection Regulation 2018.

The inclusion criteria were:

- Confirmed diagnosis of COVID-19 on RT-PCR;
- Availability of chest CT obtained in the emergency setting;
- Availability of outcome data;
- Availability of ventilation modality data.

Exclusion criteria were:

- Poor quality CT images due to motion artifacts or other artifacts that could impact image quality;
- Undetermined diagnosis;
- Patients transferred to other centers for scarcity of resources that could interfere with the outcome.

**Fig. 1:**
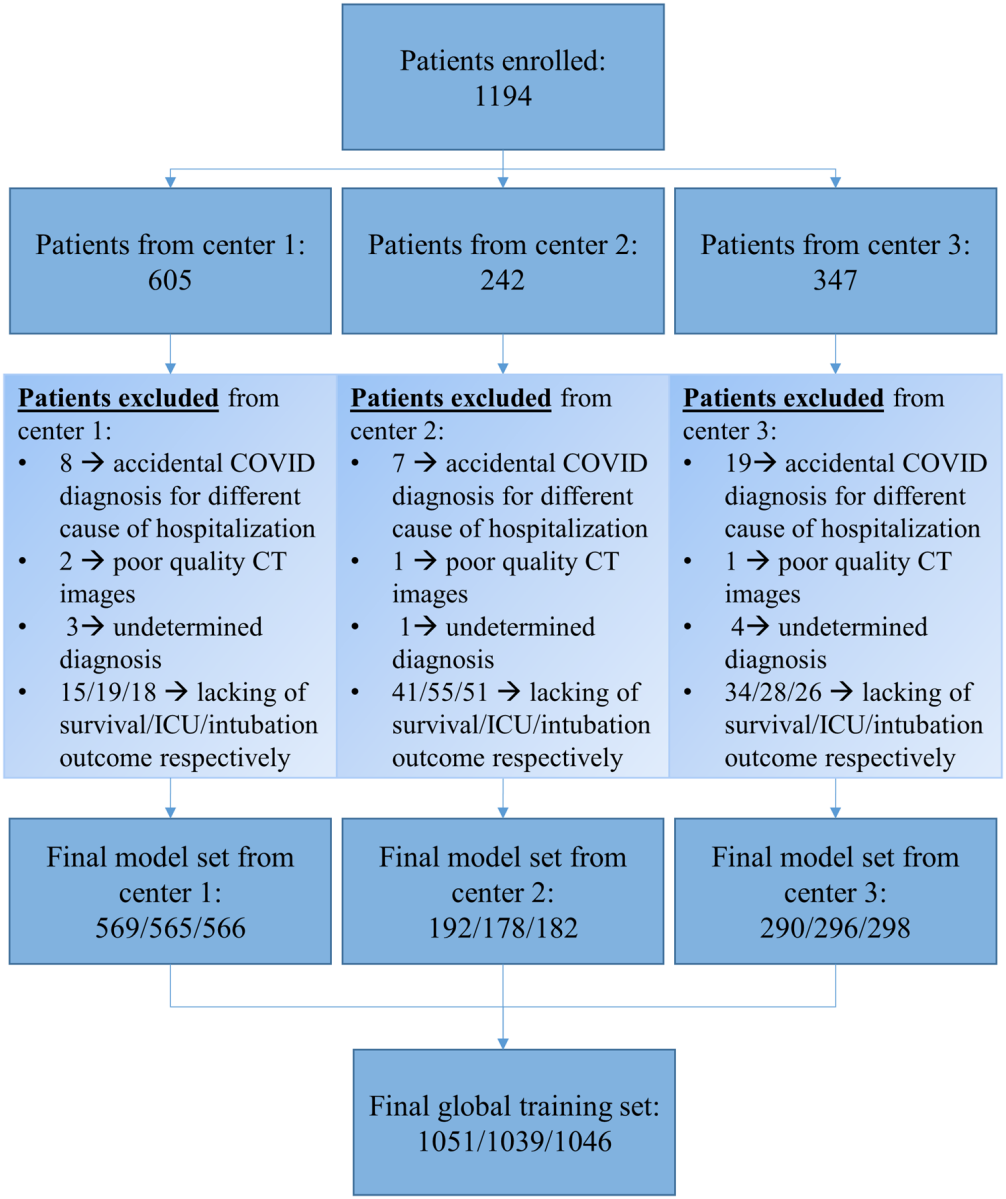
Flow diagram starting from enrolled patients to the final model set of patients. A number of samples are expressed as follows: n° of patients with mortality outcome/n° of patients with ICU outcome/n° of patients with intubation outcome

See Fig. 1 for further details.

### Clinical and Laboratory Data

Patients’ clinical records were evaluated to assess clinical and laboratory data. We evaluated signs and symptoms at presentation including fever (body temperature > 37.5 °C), dyspnea, cough, ageusia, anosmia, headache, chest pain, fatigue, arthralgia, and gastrointestinal symptoms. Comorbidities were considered as follows: hypertension, diabetes, heart disease (previous myocardial infarction, atrial fibrillation, heart failure, others), chronic obstructive pulmonary disease (COPD), chronic lung failure, cerebral vasculopathy, cancer, immunodeficiency (acquired or congenital), chronic renal insufficiency, obesity, and others (including relevant comorbidities not specified in the previous categories). We collected the following laboratory data acquired at emergency department admission: C-reactive protein, D-dimer, erythrocyte sedimentation rate, fibrinogen, white blood cells and lymphocytes level, platelets, international normalized ratio (INR), partial thromboplastin time all considered as continuous values.

Patient’s outcome was considered a binary variable at the time of the analysis (alive or deceased). Patient intensive care unit (ICU) recovery and need for intubation were also evaluated as binary outcome variables (ICU/non-ICU; intubated/not intubated).

### Imaging Acquisition and Evaluation

Images were evaluated by radiologists in each center (ADN, AB, IPV, PF) with the aim of assessing image quality and looking for COVID-19-induced alterations. Ground-glass opacities, interstitial thickening (including crazy paving), lung consolidation, and pleural effusion were considered among the CT findings of COVID-19, as done in previous studies [15–17]. We included negative chest CT as well, if the patient was hospitalized, for the sake of model generalizability.

### Imaging Pre-processing

The CT volumes were obtained for all the enrolled patients in DICOM format. To extract lung parenchyma from chest CT slices, we installed a pre-trained model from a GitHub project (https://github.com/JoHof/lungmask), based on U-net architecture for segmentation of anatomic structures [18]. After this implementation, the mask was applied on the CT slices covering the lung parenchyma volume. Due to computational cost issues, all volumes where resampled to a final volume of sixteen slices by the use of a Nibabel processing function, which resample each input voxel with *x*, *y*, and *z* dimensions to output voxel with *x*’, *y*’, and *z*’ dimensions. In particular, *x*’ = *x* and *y*’ = *y*, while *z*’ was obtained by rescaling the *z* voxel dimension to obtain a determined lower number of output slices [19], as shown in the formula:

$${z}^{^{\prime}}=\frac{{Dim}_{z}\times z}{N-1}$$

in which Dim_z_ corresponds to the *z*-axes dimension of the entire input image and *N* the number of slices to obtain as output.

**Fig. 2:**
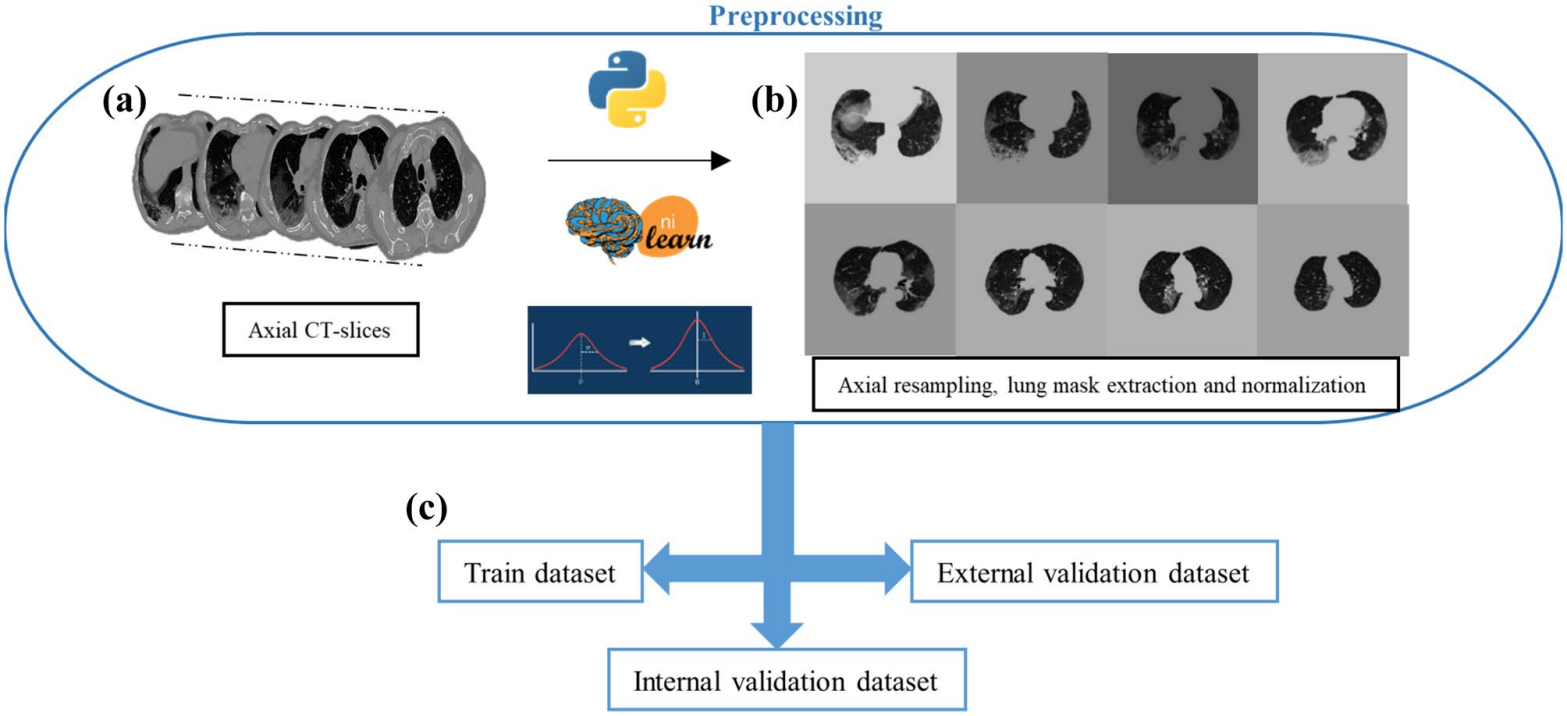
Preprocessing steps: **a** starting from CT volumes acquisitions, **b** axial resampling to eight final slices, lung mask extraction, and intensity normalization were computed before **c** training and validation on both internal and external dataset

The output image slices were finally reduced to eight after removing the slices that did not contain useful information for model training. In particular, slices with effective CT pixels’ counting and CT background pixels’ counting ratio over 70% were deleted. These steps are summarized in Fig. 2, including an example of the final patient’s volume. To rapidly processing and analyze such a large dataset, and to unify the size of CT slices, we rescaled all of them to 256 × 256 pixels, avoiding distortion by rearranging the elements of the affine matrix based on the initial and final dimensions of the axial slice. The Nilearn and Nibabel [19] libraries were adopted for CT slice pre-processing and the entire code was written in Python v.3.8 [20].

### Neural Network Architecture

**Fig. 3:**
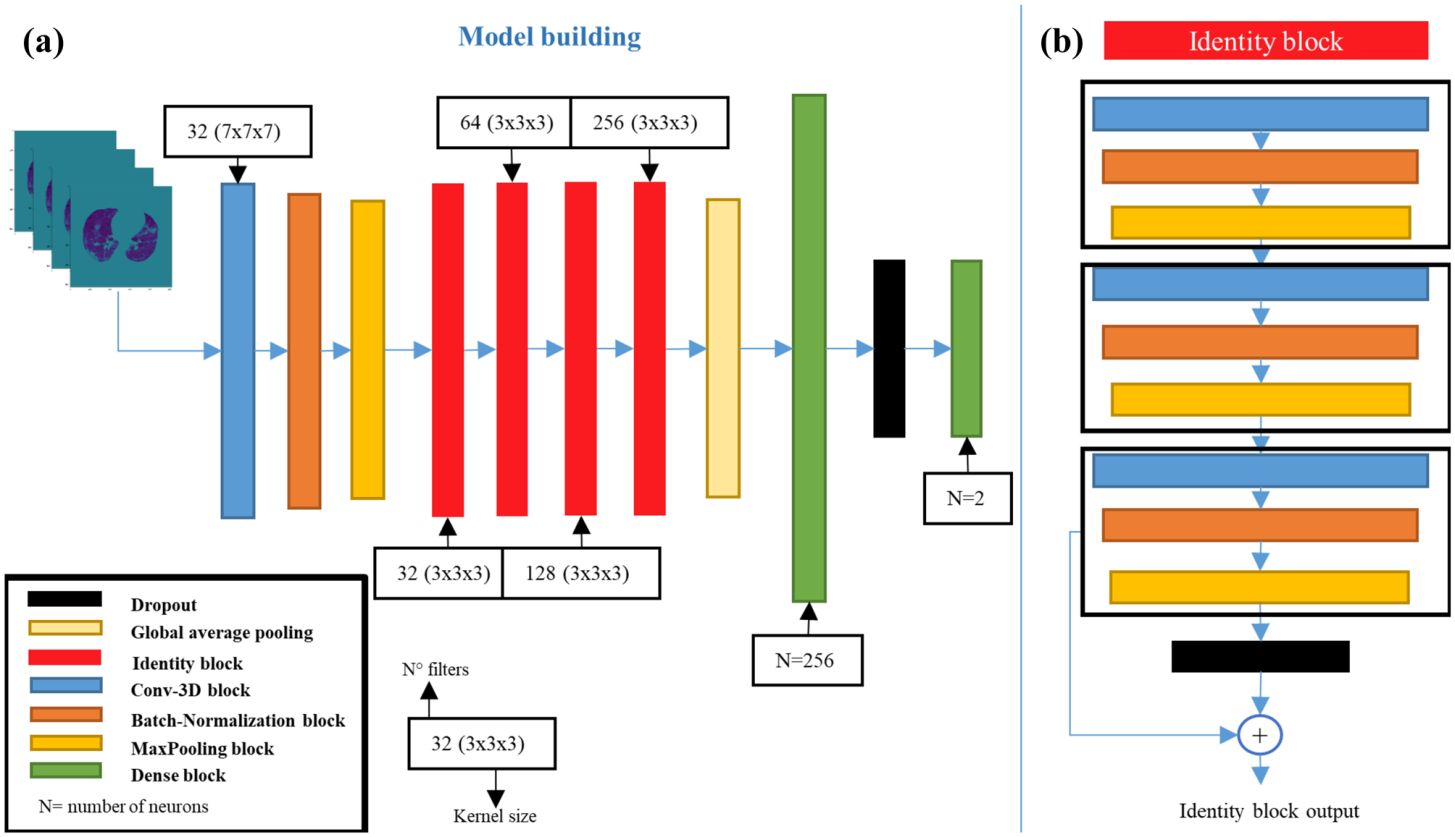
**a** 3D ResNet architecture block diagram; **b** identity block explication

We used a 3D residual neural network (3D-ResNet) (Fig. 3a) with an input layer of 256 × 256 × 8 × 1 dimensions; in each convolutional neural network (CNN) framework, there was a 3D convolution layer followed by batch normalization and max pooling layer, the latter with a pull size of 3 × 3 × 3 (considered the 3D convolution block), then four identity blocks, each one characterized by two 3D convolution blocks and a shortcut (see Fig. 3b). Finally, a global average pooling precedes the dense layer of 256 neurons, the dropout layer, and the final output layer. The convolutional layers were used for feature extraction, and a rectified linear unit (ReLU) function was used to activate the outcome of neurons. In the output layer, two softmax nodes were set to calculate two probability scores of each task with an input CT volume. Since the deceased, ICU, and intubated classes represented, respectively, 29%, 14%, and 12% of the entire CT group dataset; 28.7%, 14%, and 12% of the entire CT group dataset integrated with clinical information; and 28%, 13.5%, and 12% of the entire CT group with clinical information including laboratory data, we considered our groups unbalanced. Due to imbalance issues, we decided to assign an importance weight to each instance to adapt its effect on learning. In particular, weights were assigned using the Scikit Learn library [20] that assign them according to the formula:

$${w}_{j}= \frac{{n^\circ samples}_{tot}}{n^\circ classes\times {n^\circ samples}_{j}}$$

where *w*_*j*_ is the weight value for the class j (0/1), *n° samples*_*tot*_ is the total amount of samples including training and test, *n° samples*_*j*_ is the total amount of samples of the class j (0/1), and *n° classes* is the number of classes to predict. Due to the retrospective nature of the study, not all patients had the same outcomes to be investigated for prediction. Patients from two centers were used as training and testing groups, and patients from the third hospital served as external validation.

First, only CT images were fed to the model, then we analyzed two sub-groups of patients: one with clinical features alone and the other one with the addiction of laboratory data. We considered the model performance on the testing group as an internal validation. A randomly split into ten set of training dataset and test dataset was performed with a size ratio of 4:1, as previously reported. For each set, we choose a different randomization and patients’ shuffling was implemented. The COVID-19 patients from the third hospital were used as external independent validation of the model. Parameter optimization was implemented including different combination of the number of learning filters for each convoluted layer; several optimizers and learning rates were tested (Supplementary Table 1). The accuracy (ACC), sensitivity (SENS), and specificity (SPEC) positive and negative predictive values (PPV, NPV), Matthew Correlation Coefficient (MCC), and F1-score were computed for all validation groups (training, test/internal, external).

$$Accuracy= \frac{TP+TN}{TP+TN+FP+FN}$$

$$PPV=\frac{TP}{TP+FP}$$

$$NPV=\frac{TN}{TN+FN}$$

$$Sensitivity=\frac{TP}{TP+FN}$$

$$Specificity=\frac{TN}{TN+FP}$$

$$MCC=\frac{TP\times TN-FP\times FN}{\sqrt{(TP+FP)\times (TP+FN)\times (TN+FP)\times (TN+FN)}}$$

$$F1-score=\frac{2\times PPV\times Sensitivity}{PPV+Sensitivity}$$

The receiving operator characteristic (ROC) curves were plotted (Supplementary Figures) and area under the curve (AUC) values reported. We also integrated our model with clinical information including age, sex, comorbidities, symptoms, and laboratory tests to build another less complex architecture. The clinical features with non-binary values (laboratory exams and age) were normalized according that equation:

$$F=\frac{f-{f}_{min}}{{f}_{max}-{f}_{min}}$$

where F is the normalized value of the feature; *f*, *f*_*min*_, and *f*_*max*_ are the minimum and maximum value over all patients with that feature, respectively. To investigate the influence of clinical information on model performance, we extracted the 256 neuron layer of the best 3D model previously selected and we added age, gender, laboratory tests, comorbidities, and symptoms columns. We obtained a final matrix with 282 total features (277 without laboratory tests). Before model training, we applied oversampling technique on training group to overcome imbalance issues and avoid a number of predictor parameters higher than number of participant with outcome [21]. We tested dense neural networks with different settings of layers (Supplementary Figures for details). The same parameter optimization already mentioned was implemented. The best performing model was selected according to highest ACC, SENS, and SPEC values that are indicative for single-class accuracy prediction, due to their technical definition [22]. The same metrics abovementioned were evaluated for this model giving internal and external validation. For model training, we used an Intel(R) Xeon(R) Silver 4116 central processing unit, 25 GB of RAM and two NVIDIA GeForce RTX 3090, and Keras v.2.3.2 library. During all models training, Keras Callbacks API (https://keras.io/about/) were implemented to reduce overfitting [23].

## Results

All demographics of the patient groups (patients with CT, clinical features, and laboratory data) are outlined in Table 1 and in Supplementary Table 2. Specifics regarding the group sets for each performed training can be found in Table 2.

**Table 1:** Summary of demographics, comorbidities, symptoms, and outcomes of patients admitted with SARS-CoV-2

**Demographics**	
Age	69 years (22-102)
Sex	628 M / 403 F

**Symptoms**	
Dyspnoea	797 (77.3%)
Cough	444 (43%)
Ageusia	50 (4.8%)
Anosmia	66 (6.4%)
Chest pain	96 (9.3%)
Headache	46 (4.5%)
Fatigue	228 (22%)
Arthralgia	93 (9%)
Gastrointestinal symptoms	94 (9%)
Fever (>37.5°)	515 (50%)

**Comorbidities**	
Hypertension	472 (45.7%)
Diabetes	199 (19.3%)
Heart Disease (Previous myocardial infarction)	746 (72.3%)
Heart Disease (Atrial fibrillation)	226 (22%)
Heart Disease (Hearth failure)	59 (5.7%)
COPD	120 (11.6%)
Chronic lung failure	17 (1.6%)
Cerebral vasculopathy	86 (8.3%)
Cancer	74 (7.2%)
Chronic renal insufficiency	88 (8.5%)
Immunodeficiency	19 (1.8%)
Obesity	212 (20.5%)

**Outcomes**	
Deaths	296 (28.7%)
Number of days prior to death	14 ± 12.5 (mean ± STD)
Survived	735 (71.3%)
Recovery days	20 ± 14.3 (mean ± STD)
Intubated	125 (12.2%)
Number of days intubated	20.2 ± 15.2 (mean ± STD)
ICU	144 (14%) 21 not intubated (15%)
Number of days spent in ICU	21.2 ± 17.6 (mean ± STD)

*STD* standard deviation, *ICU* intensive care unit, *COPD* chronic obstructive pulmonary disease

**Table 2:** Summary patients’ number for each group and sub-groups reported for each predicted outcomes and kind of validation

***Patients’ group***	*Outcomes*	*Dataset dimension*	*Training group*	*Test group*	*External validation group*	*Center 1*	*Center 2*	*Center 3*
*CT*	Mortality	1051 (306 deceased)	608 (211 deceased)	153 (48 deceased)	290 (47 deceased)	569	192	290
	ICU-admission	1039 (151 ICU)	594 (103 ICU)	149 (26 ICU)	296 (22 ICU)	565	178	296
	Intubation	1046 (133 intubated)	598 (89 intubated)	150 (21 intubated)	298 (23 intubated)	566	182	298
*CT* + *CF*	Mortality	1031 (296 deceased)	**784** (**392** deceased)	153 (48 deceased)	289 (46 deceased)	565	177	289
	ICU-admission	1022 (144 ICU)	**998** (**499** ICU)	146 (26 ICU)	295 (21 ICU)	552	175	295
	Intubation	1026 (125 intubated)	**968** (**484** intubated)	147 (19 intubated)	295 (21 intubated)	556	175	295
*CT* + *CF* + *LD*	Mortality	842 (237 deceased)	**606** (**303** deceased)	115 (37 deceased)	269 (45 deceased)	417	156	269
	ICU-admission	836 (113 ICU)	**758** (**379** ICU)	113 (24 ICU)	275 (20 ICU)	407	154	275
	Intubation	839 (102 intubated)	**774** (**387** intubated)	113 (18 intubated)	275 (20 intubated)	410	154	275

*CT* computerized tomography group, *CT* + *CF* computerized tomography and clinical features group, *CT* + *CF* + *LD* computerized tomography, clinical features, and laboratory data group. In bold the number of training samples after data augmentation

### Mortality Prediction

**Fig. 4:**
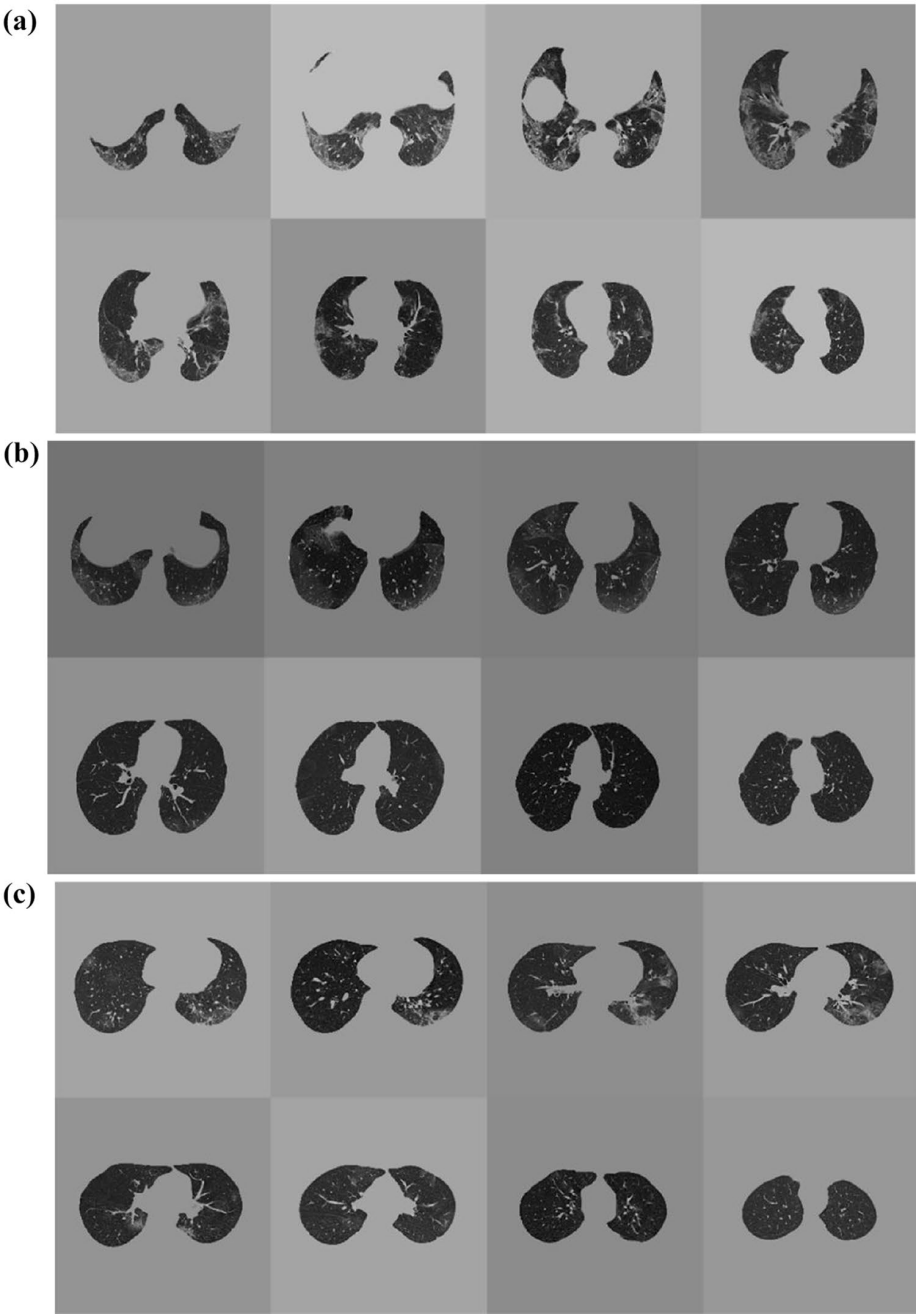
CT examples of patients correctly predicted in **a** survivor, **b** intubation, and **c** ICU admission prediction

**Fig. 5:**
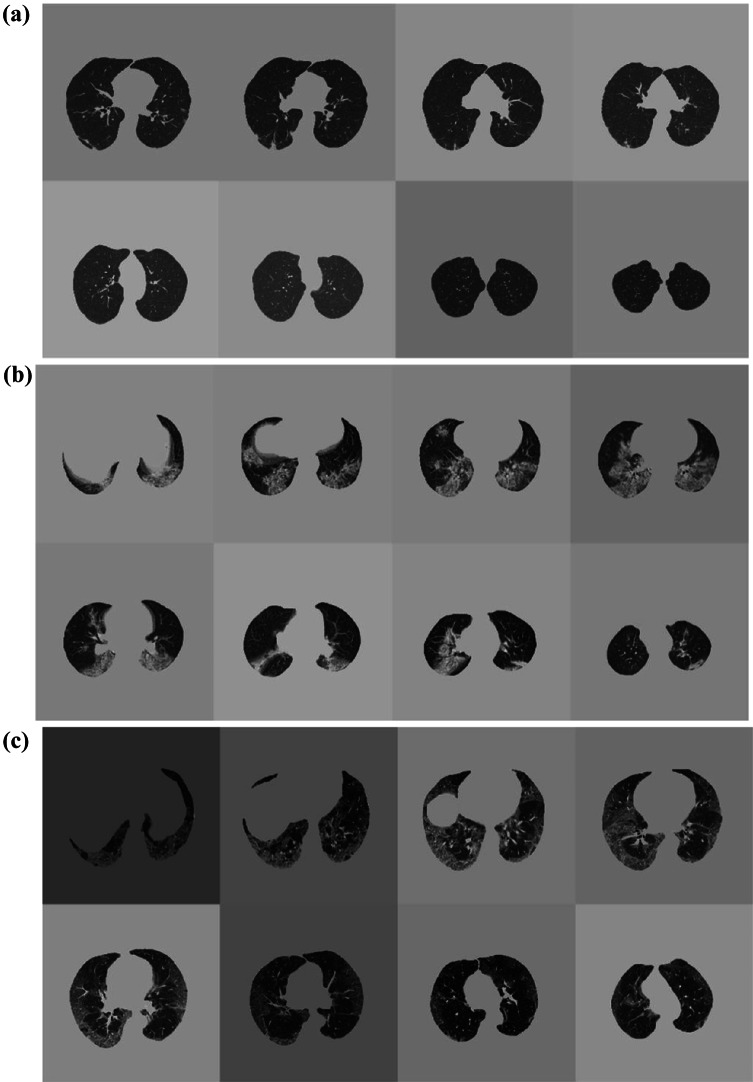
CT examples of patients not-correctly predicted in **a** survivor, **b** intubation, and **c** ICU admission prediction

DL evaluation results are outlined in Table 1. CT examples of correctly and non-correctly predicted outcomes are shown in Figs. 4 and 5.

The analysis with CT images alone resulted in accuracy of 68.3%, sensitivity of 28%, specificity of 89.7%, and PPV and NPV of 70.5% and 59.3% respectively; results for external validation are as follows: accuracy of 80.4%, sensitivity of 28.7%, specificity of 90.4%, PPV and NPV of 86.8% and 38%. Adding sex, age, symptoms, and comorbidities to our model led to the following results: accuracy 91.7%, sensitivity 90.5%, specificity 92.4%, PPV and NPV 94.8% and 86.2%; the external validation results were as follows: accuracy 71.73%, sensitivity of 55.6%, specificity of 74.8%, PPV and NPV of 90% and 29.8% (see Table 3 for other metrics evaluated).

**Table 3:** Summary results reported for each predicted outcomes and kind of validation

***Outcomes***	*AI model*	*Validation*	*ACC*	*SENS*	*SPEC*	*PPV*	*NPV*	*ROC-AUC*	*MCC*	*F1-score*
*Mortality*	CT	Internal	68.3%	28%	89.7%	70.5%	59.3%	77%	0.23	40%
		External	80.4%	28.7%	90.4%	86.8%	38%	70%	0.22	43%
	CT + CF	Internal	91.7%	90.5%	92.4%	94.8%	86.2%	95%	0.82	92.6%
		External	71.7%	55.6%	74.8%	90%	29.8%	72%	0.25	68.7%
	CT + CF + LD	Internal	92.7%	90.5%	93.7%	95%	88.7%	96%	0.84	92.7
		External	70.2%	66%	71%	91.2%	31.6%	74%	0.3	76.6%
	CF + LD	Internal	66%	53.7%	72.5%	75%	50.8%	71%	0.26	62.6%
		External	76.8%	11.5%	89%	84%	16%	45%	0.005	20.2%
*Intubation*	CT	Internal	70.3%	75%	41.7%	22.4%	88.6%	63%	0.14	34.5%
		External	71.9%	75.4%	30%	9.3%	92.8%	53%	0.03	16.6%
	CT + CF	Internal	91.3%	91.5%	89.8%	64.3%	98.3%	95%	0.71	75.5%
		External	72.6%	74.7%	45.7%	12.4%	94.7%	64%	0.12	21.3%
	CT + CF + LD	Internal	90%	90%	90.3%	60%	98.3%	95%	0.7	72%
		External	70.7%	72.3%	50%	12.6%	95%	66%	0.3	21.5%
	CF + LD	Internal	64%	62.6%	71.3%	28.4%	91.8%	68%	0.26	40%
		External	68.7%	71.3%	34.3%	8.8%	93.4%	51%	0.03	16%
*ICU admission*	CT	Internal	75.3%	84%	37%	38%	86.8%	73%	0.21	52.3%
		External	80%	84%	30%	15.5%	93.8%	63%	0.11	26%
	CT + CF	Internal	89.6%	90%	86.5%	65.6%	97%	94%	0.69	76%
		External	74.7%	77%	46%	13.4%	95%	70%	0.14	23%
	CT + CF + LD	Internal	89%	89%	86.7%	60.7%	97.7%	94%	0.66	72%
		External	73%	74.6%	52%	14%	95.2%	69%	0.16	24%
	CF + LD	Internal	60%	57.5%	70%	20.7%	92.4%	73%	0.19	30.4%
		External	76%	79%	36%	11.7%	94%	57%	0.09	20.4

*ACC* accuracy, *SENS* sensitivity, *SPEC* specificity, *PPV* positive predictive value, *NPV* negative predictive value, *ROC-AUC* receiving operator characteristic – area under the curve, *MCC* Matthew correlation coefficient, *CT* computerized tomography features, *CT* + *CF* computerized tomography features and clinical features except laboratory data, *AI* artificial intelligence, *CT* + *CF* + *LD* computerized tomography features, clinical features, and laboratory data, *CF* + *LD* clinical features and laboratory data

The analysis of patients with laboratory data led to a further increase in sensitivity (66%) and all metrics are reported in Table 3. We tested the impact of CT images on our model by running an analysis with only age, sex, comorbidity, and symptoms with the following results on the internal validation: accuracy 66%, sensitivity 53.7%, specificity 72.5%, PPV and NPV 75% and 50.8%, MCC 0.26, F1-score 62.6%. While the external validation of the same analysis produced the following results: accuracy 76.8%, sensitivity 11.5%, specificity 89%, PPV and NPV 84% and 16%, MCC 0.005, F1-score 20.2%.

### Intubation Prediction

The analysis with only CT images led to the following results: accuracy 70.3%, sensitivity 75%, specificity 41.7%, PPV and NPV 22.4% and 88.6%. Adding sex, age, symptoms, and comorbidities to our model led to the following results: accuracy 91.3%, sensitivity 91.5%, specificity 89.8%, PPV and NPV 64.3% and 98.3%; external validation with accuracy of 72.6%, sensitivity of 74.7%, specificity of 45.7%, PPV and NPV of 12.4% and 94.7% (see Table 3 for other metrics evaluated). The analysis of patients with laboratory data led to a further increase in specificity (50%) and all metrics are reported in Table 3. We tested the impact of CT images on the internal validation of our model by running an analysis with only age, sex, comorbidity, and symptoms with the following results: accuracy 64%, sensitivity 62.6%, specificity 71.3%, PPV and NPV 28.4% and 91.8%, MCC 0.26, F1-score 40%. While the external validation of the same analysis produced the following results: accuracy 68.7%, sensitivity 71.3%, specificity 34.3%, PPV and NPV 8.8% and 93.4%, MCC 0.03, F1-score 16%.

### ICU Admission Prediction

The analysis with only CT images led to the following results: accuracy 75.3%, sensitivity 84%, specificity 37%, PPV and NPV 38% and 86.8%. Adding sex, age, symptoms, and comorbidities to our model led to the following results: accuracy 89.6%, sensitivity 90%, specificity 86.5%, PPV and NPV 65.6% and 97%; the external validation results were as follows: accuracy 74.7%, sensitivity of 77%, specificity of 46%, PPV and NPV of 13.4% and 95% (see Table 3 for other metrics evaluated). The analysis of patients with laboratory data led to a further increase in specificity (52%) and all metrics are reported in Table 3. We tested the impact of CT images on the internal validation of our model by running an analysis with only age, sex, comorbidity, and symptoms with the following results: accuracy 60%, sensitivity 57.5%, specificity 70%, PPV and NPV 20.7% and 92.4%, MCC 0.19, F1-score 30.4%. While the external validation of the same analysis produced the following results: accuracy 76%, sensitivity 79%, specificity 36%, PPV and NPV 11.7% and 94%, MCC 0.09, F1-score 20.4%.

## Discussion

The COVID-19 pandemic represents an ideal scenario for AI applications. The need of rapid diagnosis and effective allocation of the available resources to guarantee the best treatment possible [5] opens the way for taking advantage of predictive models to optimize patient care in this critical scenario. As a consequence, numerous AI-powered predictive models have populated the literature in the past 2 years [6, 13, 14]. However, strong reproducibility and generalizability across different patient populations and different centers are needed to translate theoretical models into the clinical practice. Recent studies have advocated about the importance of standard guidelines and quality assessment of AI studies in radiology [24, 25]. External validation appears to be an important step in AI research, representing the pillar for widespread clinical application [26]. To our knowledge, the present study represents the first attempt in building a DL 3D CT-inclusive prediction model on COVID-19 patients and validating the results with internal and external validation, including patients from multiple centers and different scanners. In particular, training and test were performed on one vendor scanners in two different centers; external validation was performed on a second vendor scanner in the third center.

Our model succeeded in predicting mortality, ICU admission, and intubation with a remarkable accuracy of 91.7%, 91.3%, and 89.6% when age, sex, symptoms, and comorbidities were added to the analysis. However, the model accuracy for the same outcomes dropped significantly in the external validation cohort: mortality 71.73%, ICU admission 74.7%, and intubation 72.6%.

Chest CT is a useful exam, although not routinely administered at presentation, in COVID-19 patients to assess lung involvement, possible complications, and sometimes as a diagnostic test for its high sensitivity to recognize interstitial pneumonitis [7, 10, 27]. Chest CT correlates with clinical severity [12, 28], especially when a quantitative approach is applied [29, 30], which makes it a potential candidate to support COVID-19 predictive models. In fact, CT-based DL models have been proven effective to distinguish COVID-19 subtypes and other types of pneumonia [13, 31]. CT imaging is expected to contain valuable information for patient’s outcome, thus to serve as a base for AI analysis. However, even though numerous studies used AI models to predict patients’ outcome [6], only two included chest CT images and DL methods [32, 33]. Fang et al. developed an artificial neural network to perform lung lobes and pulmonary opacities segmentation, which served to compute a severity score and predict mortality through another machine-learning algorithm. The model achieved moderate accuracy, with an AUC of 0.74 [32]. Similarly to our research, Ning et al. created a DL model based on a single, manually segmented, 2D chest CT slice for mortality prediction of COVID-19 patients taken from two different hospitals, with reported accuracy of 76.41% [33]. This result was improved by integrating the model with laboratory data (78.73%) [33]. For the same reasons, we aimed at building an integrated model with imaging and clinical data to boost performance. Due to data imbalance, we opted for adding other clinical information such as age, sex, comorbidities, and symptoms at presentation, which led to superior results in mortality prediction in the internal validation cohort: accuracy 91.7%, sensitivity 90.5%, specificity 92.4%. It is known, in fact, that certain comorbidities are associated with increased risk of mortality [34]. Nevertheless, we decided to analyze the sub-group of patients with available laboratory data. Although the reduced number of patients and the above cited imbalance, and although the introduction of a different type of values (continuous vs. binary), the performance in mortality prediction remained elevated in the internal validation cohort (accuracy 92.8%, sensitivity 91.6%, specificity 93.3%). These results could underlie an important role of laboratory data in outcome prediction. Nonetheless, it is important to remark the strong imbalance in the dataset as one of the limitations of the study since a proper comparison between the analysis with and without laboratory data cannot be made. Other studies reported the impact of symptoms, comorbidities, and CT abnormalities as predictors for hospitalization and need of mechanical ventilation [6, 35]. Similarly, our results improved significantly when all these information were considered. Furthermore, when CT images were excluded from the model, the accuracy dropped impressively to 66%, 64%, and 60% in predicting mortality, ICU admission, and intubation. This result implies that CT imaging contains highly valuable data on patient’s status and should not be excluded from the algorithm implementation.

Chieregato et al. recently reported a hybrid machine learning/deep learning predictive model for COVID-19 based on CT images, laboratory, and clinical data [15]. The authors achieved similar results to our study in the prediction of COVID-19 outcome, without performing an external validation of their accuracy values. Despite achieving promising results in the preliminary analyses, the performance of our model for all the outcomes decreased in the external validation cohort. The most significant decrease was seen in specificity and NPV. The performance dropout can be partially explained with the heterogeneity of the population and scanning techniques, which is in line with the normal setting of patient care in real-life scenarios. On the other hand, the result points to limited generalizability. This aspect appears a crucial limitation of many currently available AI models, which affects their potential use in the clinical practice [36].

In a pandemic setting, hospital overcrowding, paucity of ICU beds, and ventilation devices represent the main challenges for resource managing [37], often leading to critical decision-making [5, 38]. Similarly to previous quantitative CT analysis studies [39, 40], our model could predict ICU admission and need for intubation with very high accuracy, sensitivity, and specificity when tested internally (89.6% and 91.3%, 90% and 91.5%, and 86.5% and 89.8%, respectively). However, the performance was not equally reproduced in the external validation. AI models may offer some advantages in a critical setting compared to quantitative evaluations performed by radiologists, due to the promise of delivering rapid and operator-independent results. Nevertheless, such advantages can be overshadowed by limited reproducibility and generalizability across different centers and patient populations, which is a pre-requisite for clinical use. In this respect, one of the future challenges is the standardization of DL algorithms across multiple centers and their validation with prospective data to achieve adequate predictive accuracy for meaningful clinical application [39, 40].

This study has some limitations. Due to retrospective design, we encountered imbalance on different outcomes caused by sporadic lack of information and transfer to other facilities. To compensate this shortcoming, part of the missing data was gathered through directly contacting patients or relatives. One of the greatest imbalances of the present research was the lower representation of deceased patients compared to the ones who survived, although previous studies encountered similar issues [32, 33]. In a retrospective study, laboratory data are usually imbalanced. Consequently, as stated before, our sub-group analysis cannot be properly compared with the whole cohort. Future directions include reproducing our results in larger and more uniform cohorts. Another consideration should be done on the different timing of presentation at the hospital after the positive on RT-PCR result (range 0–9 days; mean 5 days), which could have affected severity of CT findings; we deliberately did not consider this variable since we meant to address real-world problems, recreating an everyday pandemic setting. The images of the external validation cohort were acquired with a scanner of a different vendor from the training and internal validation cohorts. This fact affected model performance on the external group, confirming the need of a larger dataset of CT from all existing vendors to achieve optimal prediction results. Lastly, we decided to include pleural effusion into the analysis although, if conspicuous, it could have masked other lung alteration because we considered it as a possible finding in COVID-19 patients and it has been correlated with outcome in a recent meta-analysis [41].

## Supplementary Information

Below is the link to the electronic supplementary material.

Supplementary file1 (DOCX 3772 kb)

## Abbreviations

AI: Artificial intelligence
DL: Deep learning
COVID-19: Coronavirus disease 2019
SARS-CoV2: Severe acute respiratory syndrome coronavirus 2
ICU: Intensive care unit
CNN: Convolutional neural network
