# Supplementary material for: 3D CT-Inclusive Deep-Learning Model to Predict Mortality, ICU Admittance, and Intubation in COVID-19 Patients
Source: J Digit Imaging. 2022 Nov 30;36(2):603–16. doi: 10.1007/s10278-022-00734-4 (PMC9713092; doi:10.1007/s10278-022-00734-4)
Supplement: Supplementary file 1 — Supplementary file1 (DOCX 3772 kb) [file 10278_2022_734_MOESM1_ESM.docx]

**Supplementary Materials**

***Supplementary Methods***

**Imaging acquisition**

100-120 kVp and mAs adapted according to automatic exposure control (AEC). The field of view included the whole chest and was acquired during a single forced inspiration, in keeping with patient compliance, performed in the cranio-caudal orientation with the patient in supine position. Chest CT images were reconstructed using 1-mm slice thickness/1-mm reconstruction interval with high-spatial-frequency and soft-tissue reconstruction algorithms for the evaluation of lung parenchyma and soft tissues, respectively.

**Neural Network Architecture**

We tested dense neural networks with different settings of layers: a 6-layers dense neural network (DNN), including input layer, a block that we indicate as convoluted block that comprehend a dropout layer, dense layer with 64 learning filters, batch- normalization, another dropout layer, finally a output layer with two nodes; a 9-layers DNN, including input layer, two convoluted blocks with 128 and 64 learning filters respectively and the final layer with two output nodes; a 12-layers DNN, including input layer, three convoluted blocks with 128,64 and 32 learning filters respectively and the final layer with two output nodes (**Supplementary Figure 1**).

***Supplementary Tables***

| **Optimizer name** | **Learning rate tested** |
| --- | --- |
| RMSprop | 0.0001,0.00001,0.001,0.1 |
| Sgd | 0.001,0.01,0.1 |
| Adam | 0.0001,0.0005,0.5,0.00001,0.001,0.00146 |
| NAdam | 0.001,0.0001,0.0005,0.00001 |

**Supplementary Table 1.** Tuned optimizers parameters for each model training.

|  | **Mean (min-max)** |
| --- | --- |
| Age | 69(22-102) |
| White blood cells (WBC) | 8.42(0.81-116) |
| Lymphocytes level | 1.31(0.02-51.2) |
| Platelets | 224.54(7.8-1012) |
| International normalized ratio (INR) | 1.25(0.65-25.2) |
| Partial thromboplastin time (aPTT) | 31.14(1.11-159) |
| C-reactive protein (CRP) | 68.43(0.0-488.9) |
|  | **Number (%) - All=842** |
| Male | 512(60.8%) |
| Dyspnea | 650(77%) |
| Cough | 384(45.6) |
| Ageusia | 44(5%) |
| Anosmia | 57(6.7%) |
| Chest pain | 92(10.9%) |
| Headache | 40(4.7%) |
| Fatigue | 204(24.2%) |
| Arthralgia | 83(9.8%) |
| Gastrointestinal symptoms | 82(9.7%) |
| Hypertension | 378(44.8%) |
| Diabetes | 156(18.5%) |
| Heart Disease (Coronopathy) | 617(73.3%) |
| Heart Disease (Atrial fibrillation) | 180(21.4%) |
| Heart Disease (Hearth failure) | 45(5.3%) |
| COPD | 90(10.6%) |
| Chronic lung failure | 12(1.4%) |
| Cerebral vasculopathy | 71(8.4%) |
| Cancer | 60(7%) |
| Immunodeficiency | 14(1.6%) |
| Chronic renal insufficiency | 71(8.4%) |
| Obesity | 165(7.7%) |
| Fever (>37.5°) | 436(51.8%) |
| **Outcomes** |  |
| Deaths | 237(28%) |
|  | **Mean ± STD** |
| Number of days prior to death | 13.4 ± 12.76 |
| Survived | 605(72%) |
|  | **Mean ± STD** |
| Recovery days | 20 ± 14.4 |
|  |  |
|  | **Number (%) - All=839** |
| Intubated | 102(12%) |
|  | **Mean ± STD** |
| Number of days intubated | 19.3 ± 17.8 |
|  |  |
|  | **Number (%) - All=836** |
| ICU | 113(13.5%) |
|  | **Mean ± STD** |
| Number of days spent in ICU | 21 ± 17.4 |

**Supplementary Table 2.** Summary of demographics, comorbidities, symptoms, laboratory data and outcomes of patients’ sub-group admitted with SARS-CoV-2.

***Supplementary Figures***

**
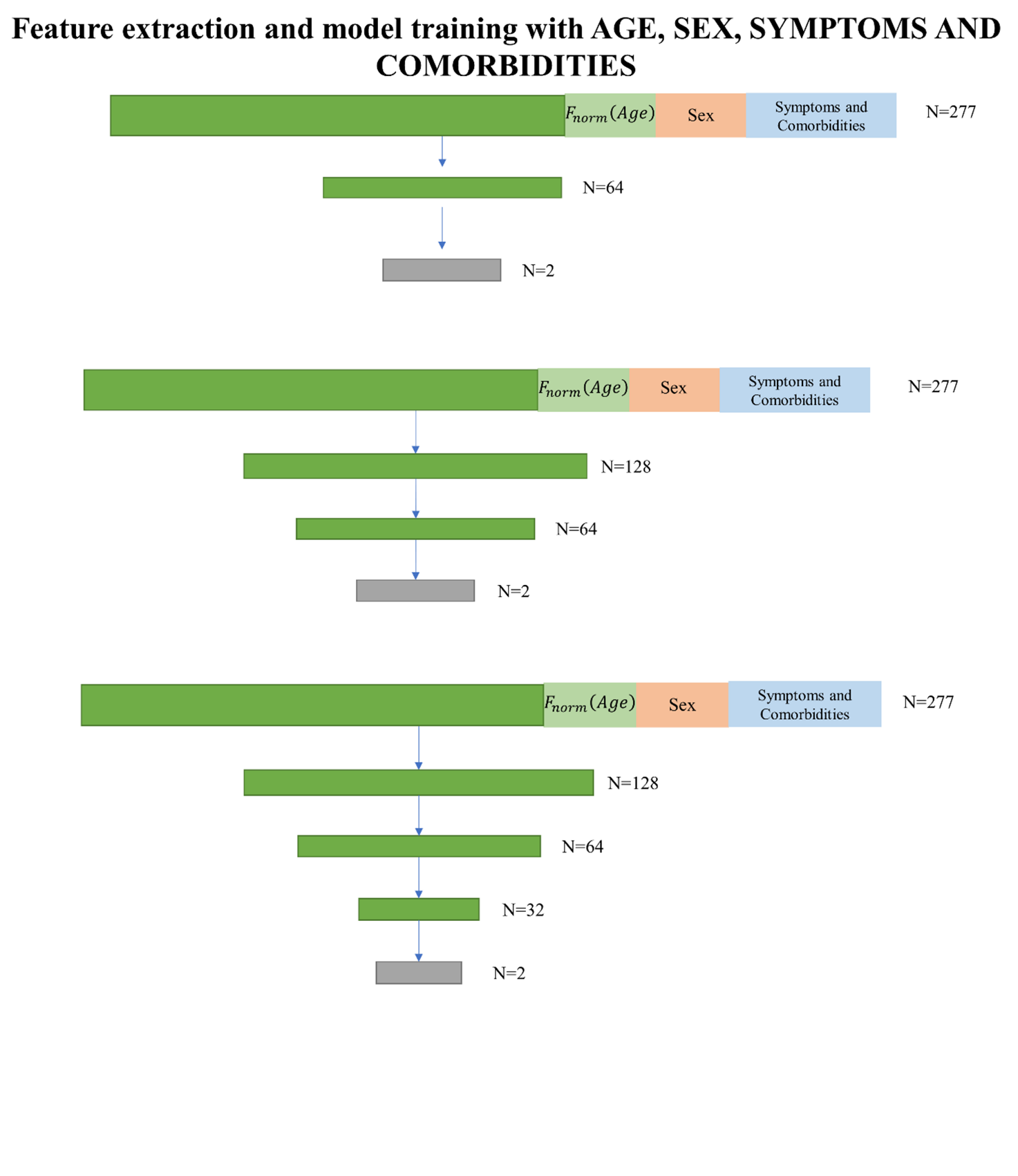
**

**Supplementary Figure 1.** The three customized DNN used for retraining of CT extracted features together with normalized age, sex, symptoms and comorbidities. N= number of neurons in each layer. DNN= dense neural network; CT= computerized tomography


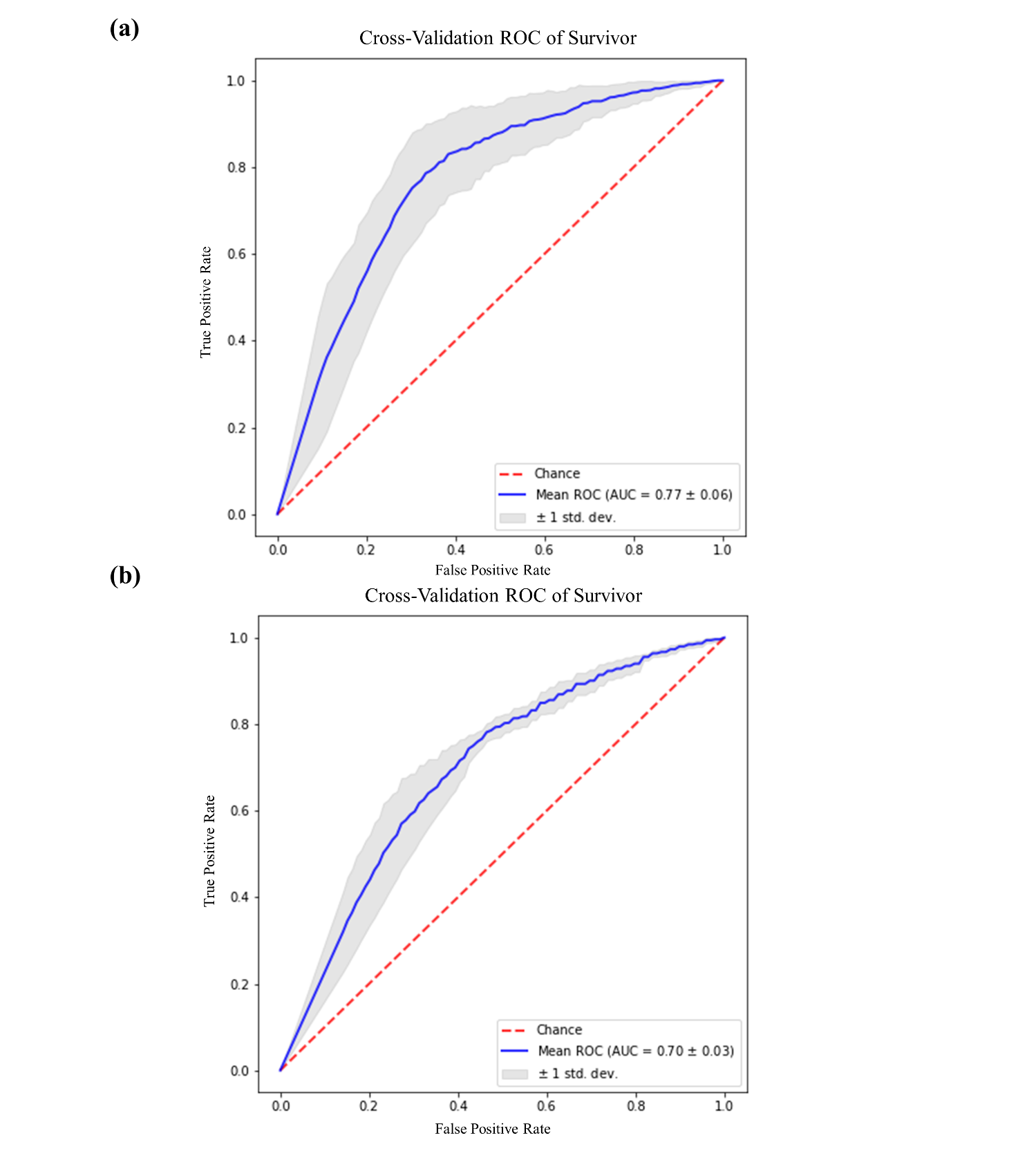


**Supplementary Figure 2.** ROC-AUC plot of survivor prediction model with only CT features and implementing **(a)** internal and **(b)** external validation. ROC-AUC= receiving operator characteristic – area under the curve; CT= computerized tomography


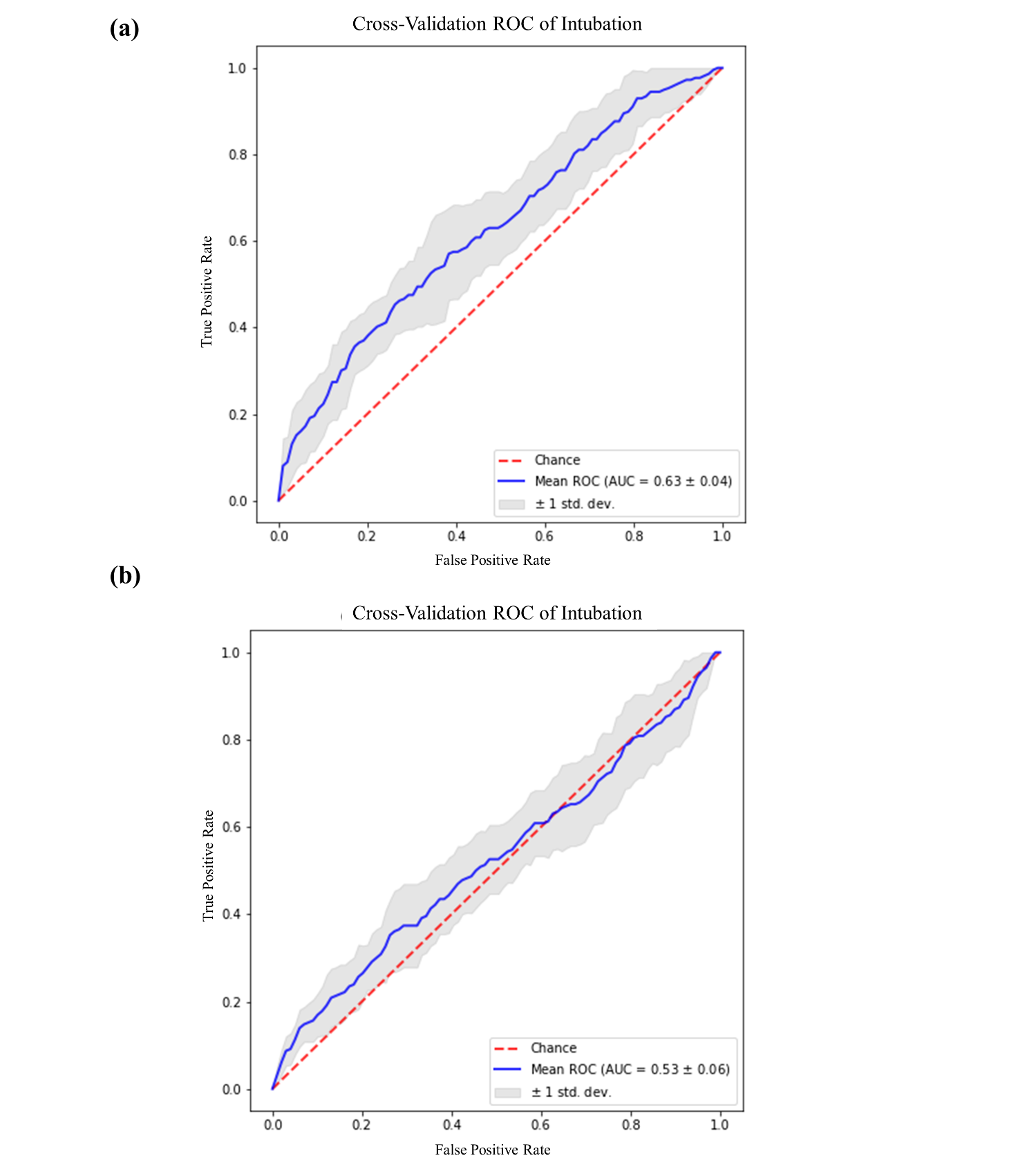


**Supplementary Figure 3.** ROC-AUC plot of intubation prediction model with only CT features and implementing **(a)** internal and **(b)** external validation. ROC-AUC= receiving operator characteristic – area under the curve; CT= computerized tomography


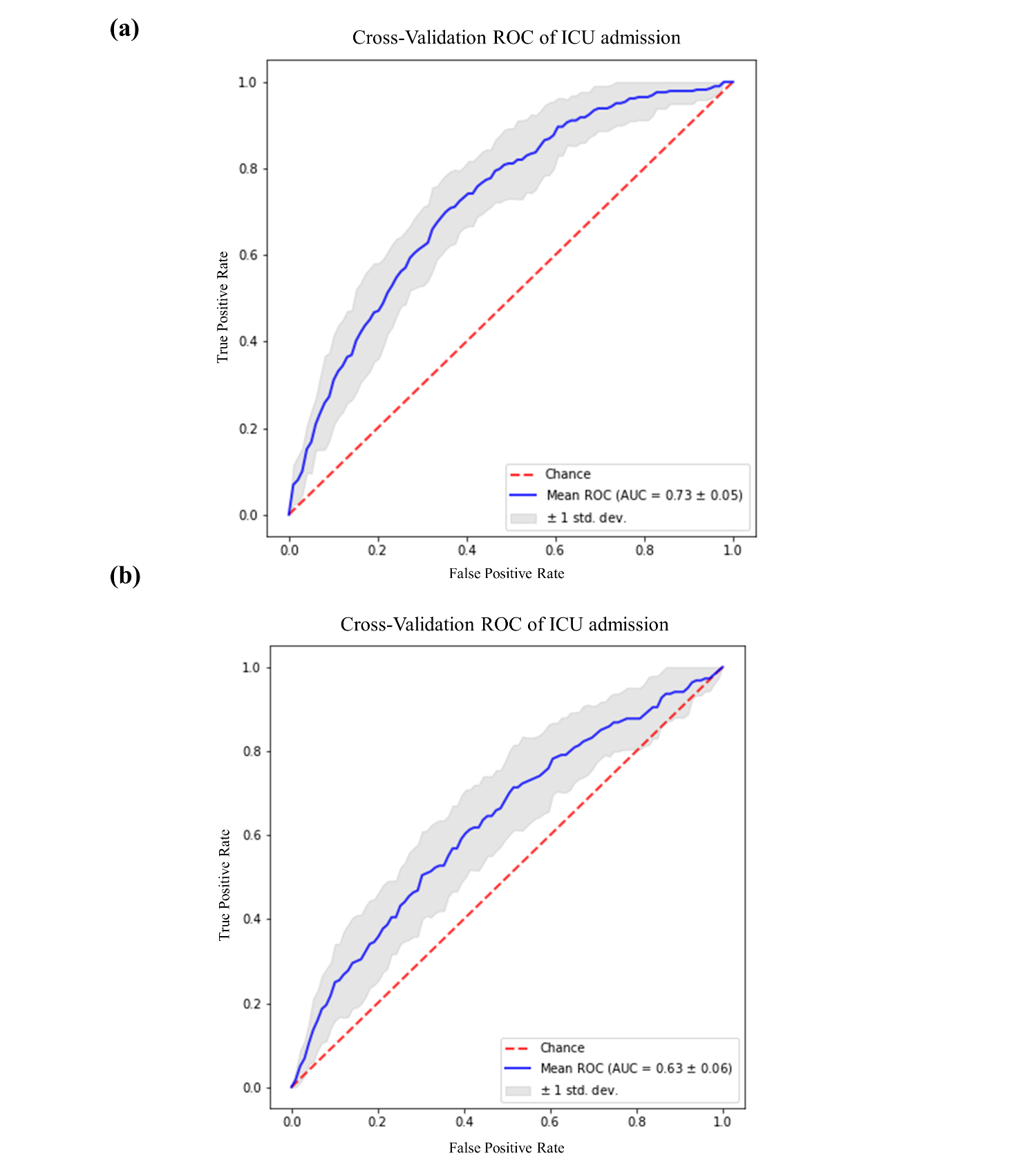


**Supplementary Figure 4.** ROC-AUC plot of ICU admission prediction model with only CT features and implementing **(a)** internal and **(b)** external validation. ROC-AUC= receiving operator characteristic – area under the curve; CT= computerized tomography; ICU= intensive care unit


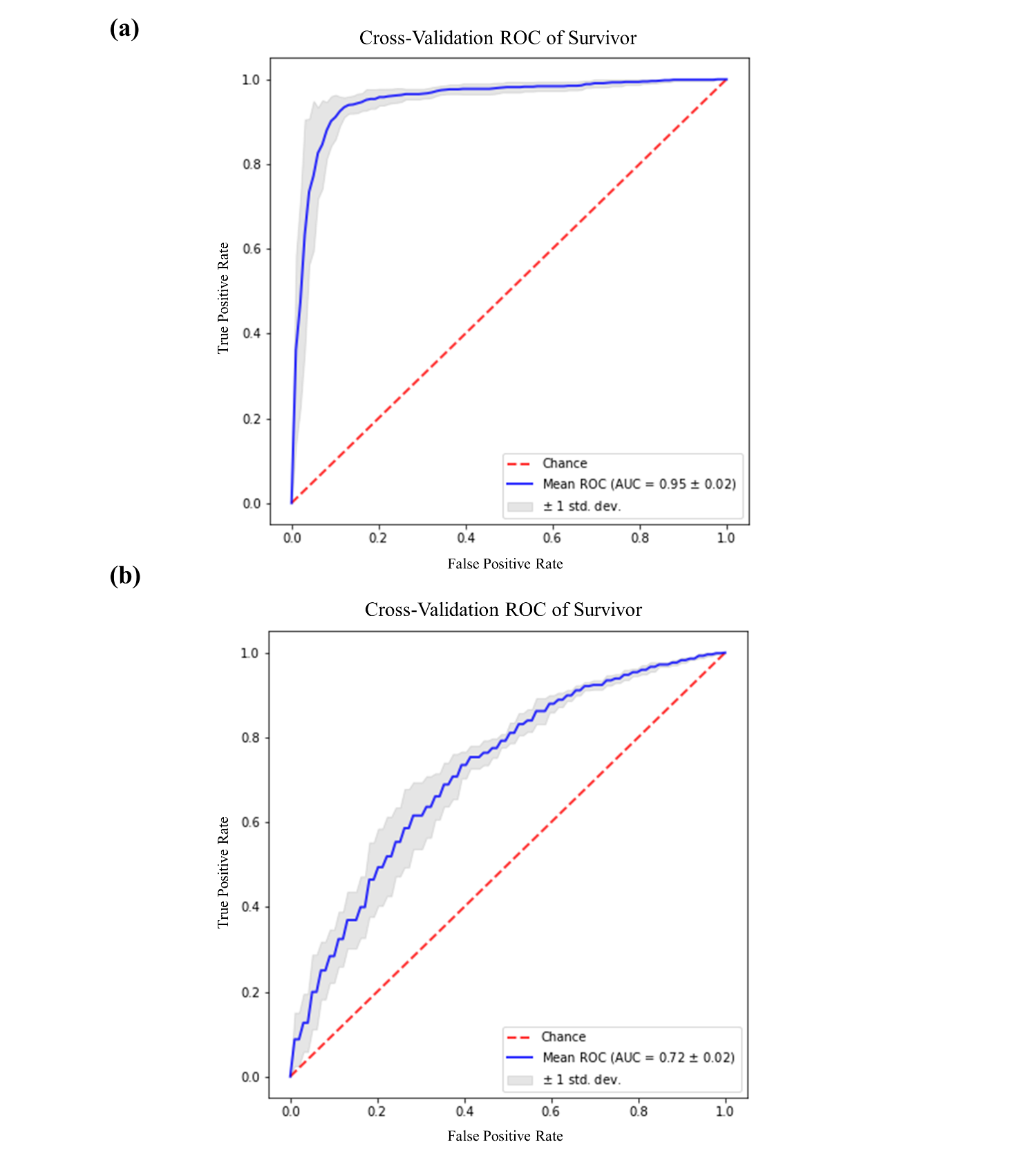


**Supplementary Figure 5.** ROC-AUC plot of survivor prediction model with retraining of augmented CT features together with age, sex, symptoms and comorbidities, implementing **(a)** internal and **(b)** external validation. ROC-AUC= receiving operator characteristic – area under the curve; CT= computerized tomography


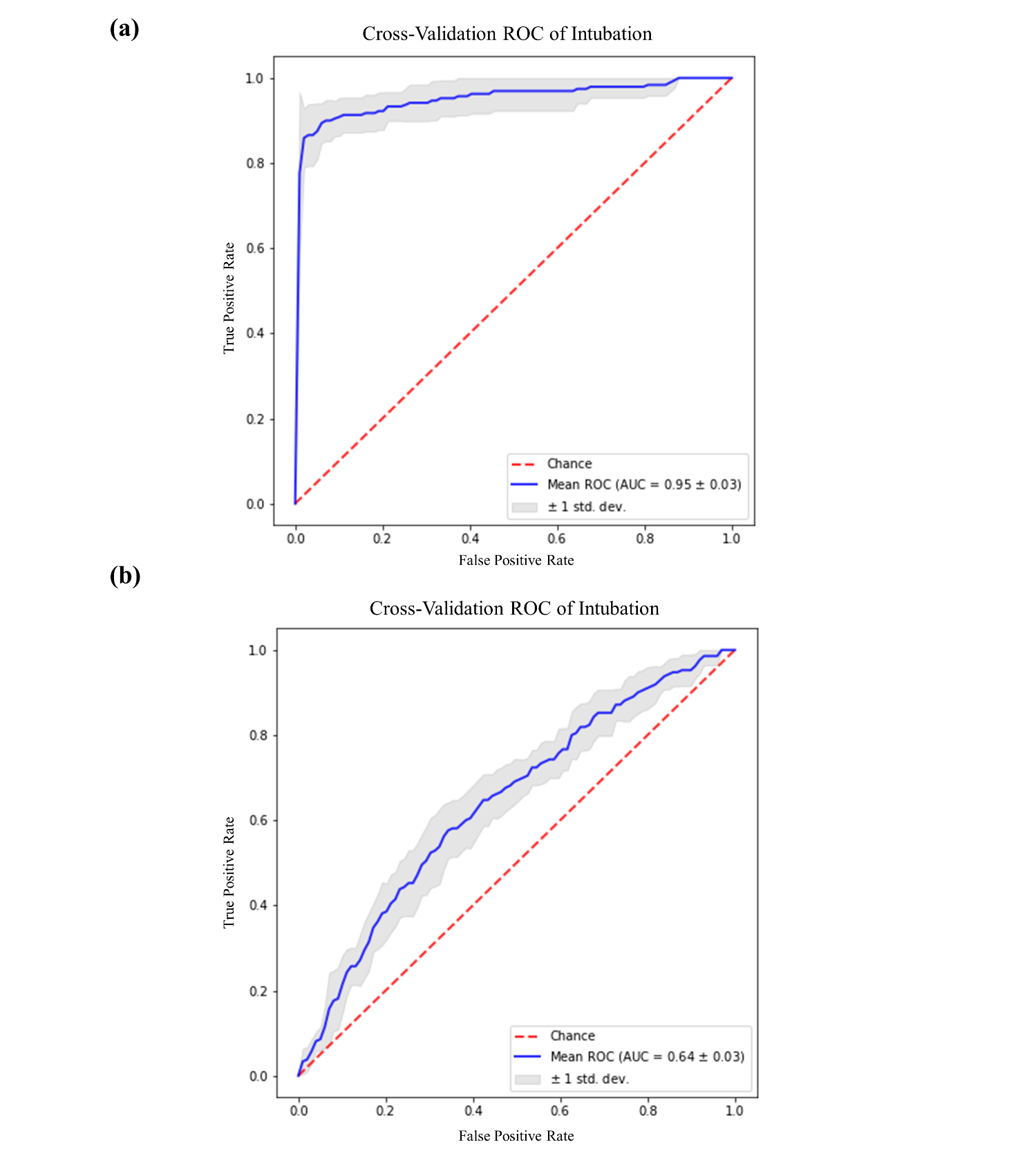
**Supplementary Figure 6.** ROC-AUC plot of intubation prediction model with retraining of augmented CT features together with age, sex, symptoms and comorbidities, implementing **(a)** internal and **(b)** external validation. ROC-AUC= receiving operator characteristic – area under the curve; CT= computerized tomography.


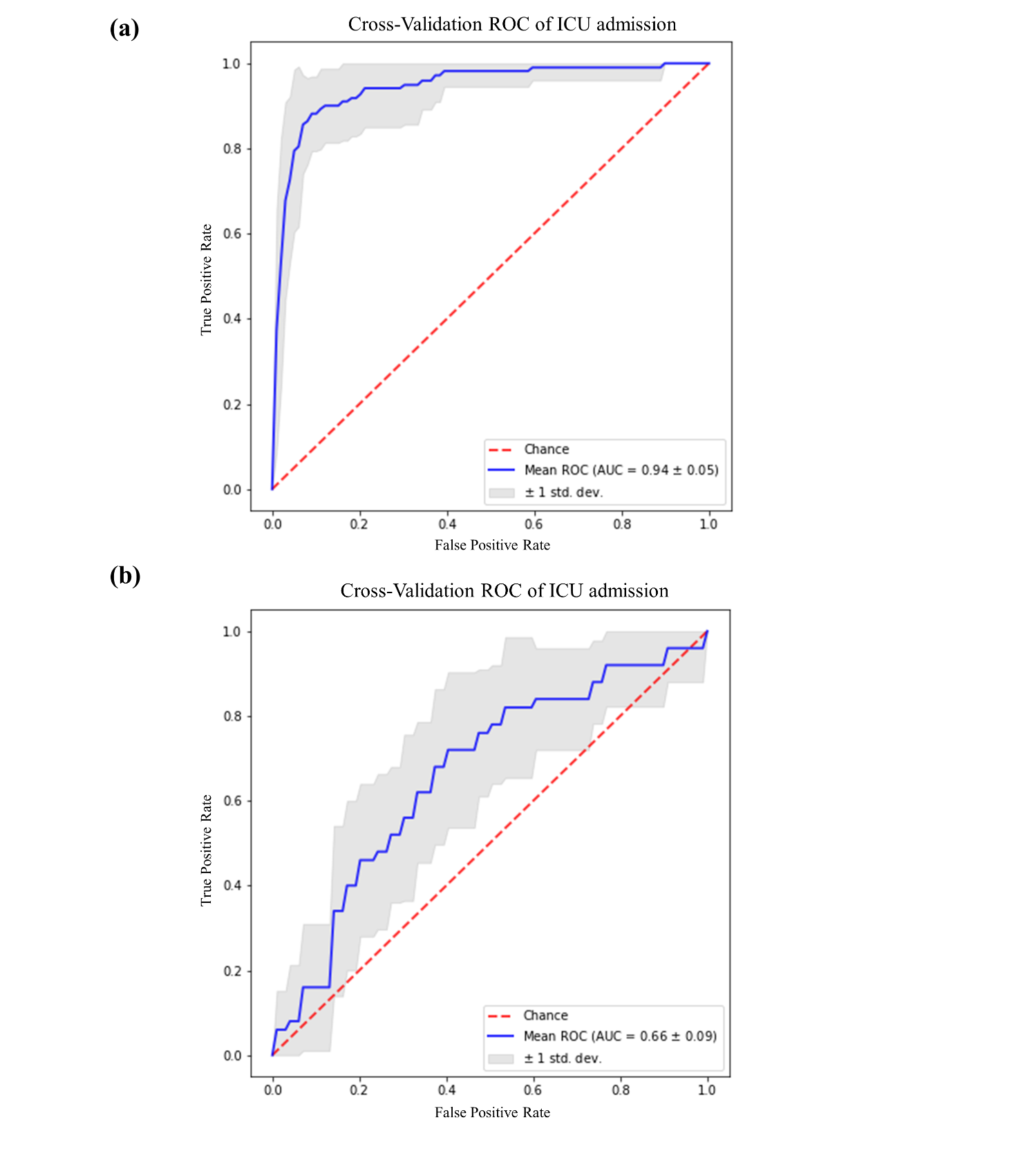


**Supplementary Figure 7.** ROC-AUC plot of ICU admission prediction model with retraining of augmented CT features together with age, sex, symptoms and comorbidities, implementing **(a)** internal and **(b)** external validation. ROC-AUC= receiving operator characteristic – area under the curve; CT= computerized tomography; ICU= intensive care unit.


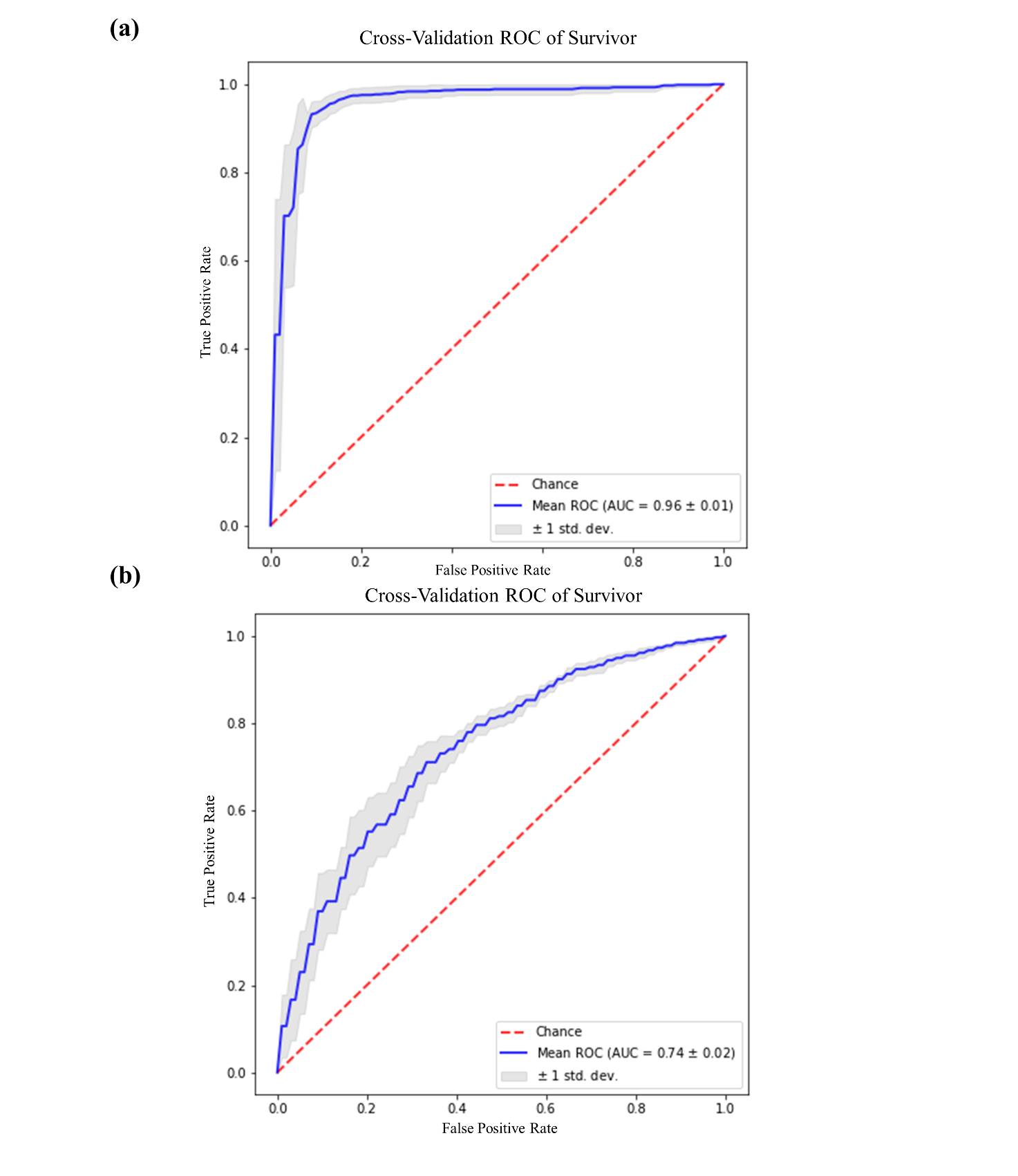


**Supplementary Figure 8.** ROC-AUC plot of survivor prediction model with retraining of augmented CT features together with age, sex, symptoms, comorbidities and laboratory data, implementing **(a)** internal and **(b)** external validation. ROC-AUC= receiving operator characteristic – area under the curve; CT= computerized tomography.


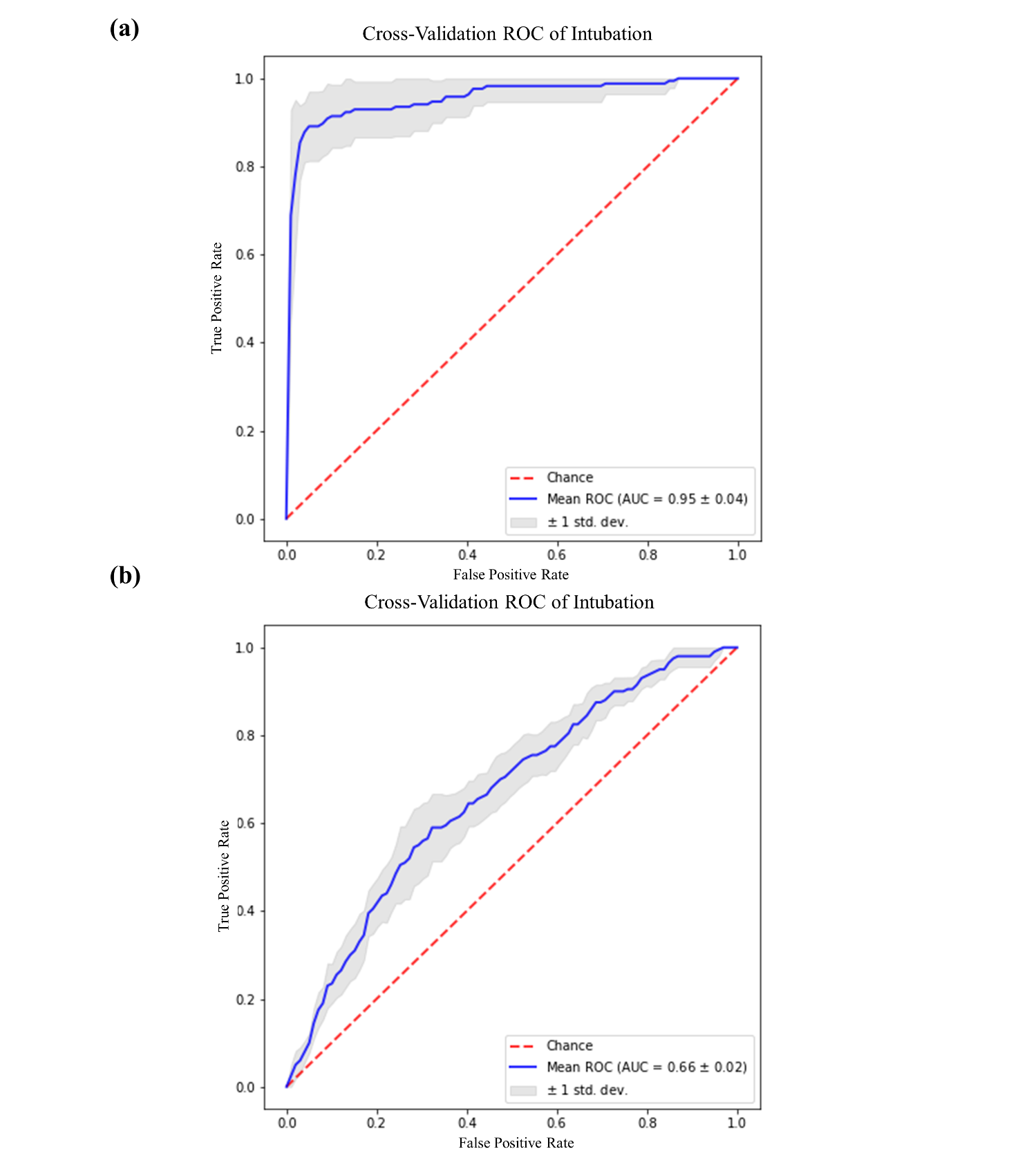


**Supplementary Figure 9.** ROC-AUC plot of intubation prediction model with retraining of augmented CT features together with age, sex, symptoms, comorbidities and laboratory data, implementing **(a)** internal and **(b)** external validation. ROC-AUC= receiving operator characteristic – area under the curve; CT= computerized tomography.


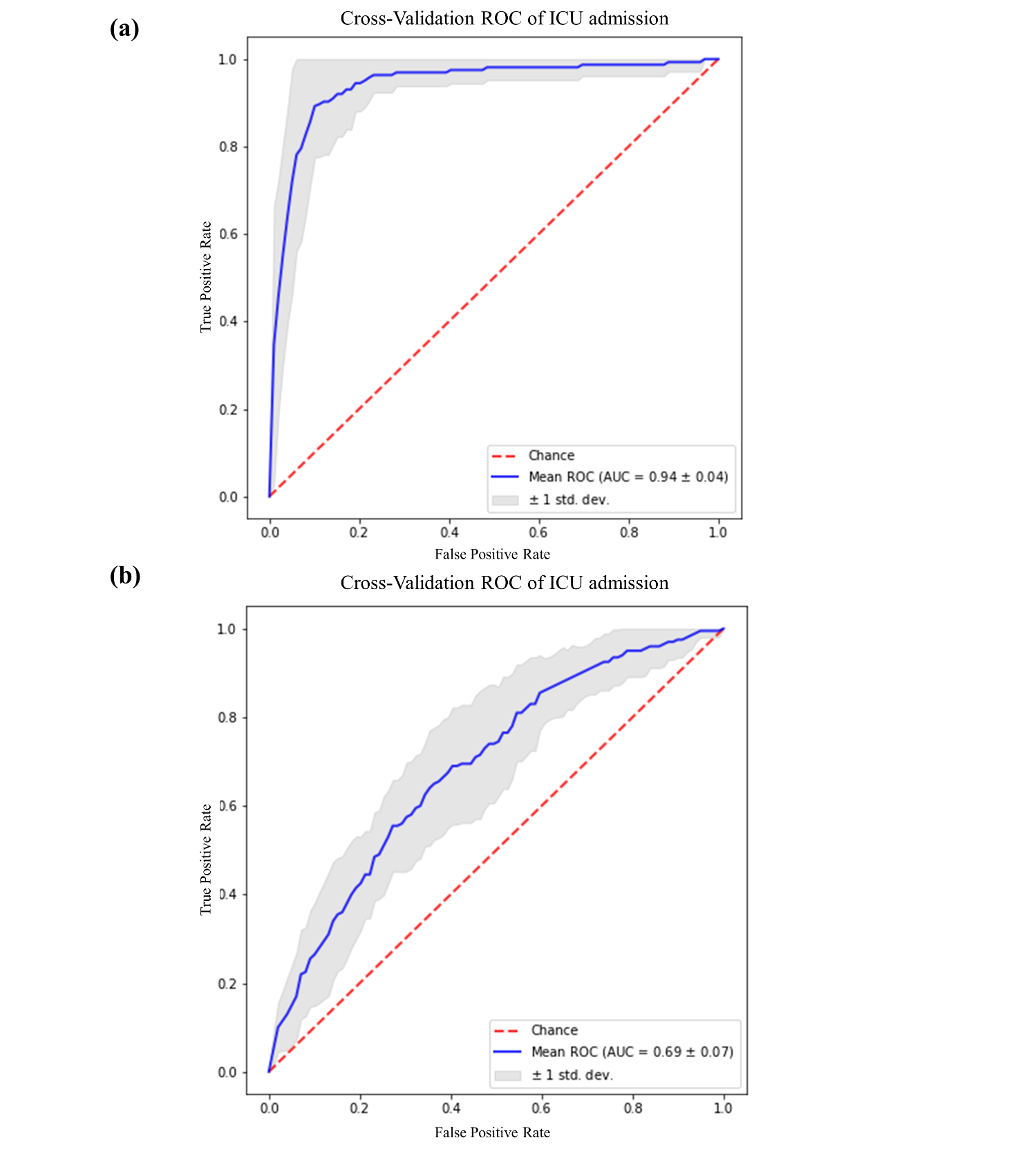


**Supplementary Figure 10.** ROC-AUC plot of ICU admission prediction model with retraining of augmented CT features together with age, sex, symptoms, comorbidities and laboratory data, implementing **(a)** internal and **(b)** external validation. ROC-AUC= receiving operator characteristic – area under the curve; CT= computerized tomography; ICU= intensive care unit.
